# Molecular Characterization of TRPA Subfamily Genes and Function in Temperature Preference in *Tuta absoluta* (Meyrick) (Lepidoptera: Gelechiidae)

**DOI:** 10.3390/ijms22137157

**Published:** 2021-07-02

**Authors:** Xiao-Di Wang, Ze-Kai Lin, Shun-Xia Ji, Si-Yan Bi, Wan-Xue Liu, Gui-Fen Zhang, Fang-Hao Wan, Zhi-Chuang Lü

**Affiliations:** 1 State Key Laboratory for Biology of Plant Diseases and Insect Pests, Institute of Plant Protection, Chinese Academy of Agricultural Sciences, Beijing 100193, China; 82101195160@caas.cn (X.-D.W.); lincsaurora@gmail.com (Z.-K.L.); 82101172334@caas.cn (S.-X.J.); bisiyan91@gmail.com (S.-Y.B.); liuwanxue@caas.cn (W.-X.L.); zhangguifen@caas.cn (G.-F.Z.); wanfanghao@caas.cn (F.-H.W.); 2 Agricultural Genome Institute at Shenzhen, Chinese Academy of Agricultural Sciences, Shenzhen 518120, China

**Keywords:** *Tuta absoluta*, *TRPA1*, *Painless*, *Pyrexia*, RNA interference, temperature preference

## Abstract

To reveal the mechanism of temperature preference in *Tuta absoluta*, one of the top 20 plant pests in the world, we cloned and identified *TaTRPA1*, *TaPain*, and *TaPyx* genes by RACE and bioinformatic analysis, and clarified their expression profiles during different development stages using real-time PCR, and revealed their function in preference temperature by RNAi. The full-length cDNA of *TaPain* was 3136 bp, with a 2865-bp open reading frame encoding a 259.89-kDa protein; and the partial length cDNA of *TaPyx* was 2326-bp, with a 2025-bp open reading frame encoding a 193.16-kDa protein. In addition, the expression of *TaTRPA1* and *TaPyx* was significantly lower in larvae than other stages, and it was significantly higher in pupae and newly emerging males for *TaPain*. After feeding target double-stranded RNA (dsRNA), the preferred temperature decreased 2 °C more than the control group. In conclusion, the results firstly indicated the molecular characterization of TRPA subfamily genes and their key role in temperature perception in *T. absoluta*, and the study will help us to understand the temperature-sensing mechanism in the pest, and will provide some basis for study of other Lepidoptera insects’ temperature preference. Moreover, it is of great significance in enriching the research progress of “thermos TRP”.

## 1. Introduction

The South American tomato leaf miner, *Tuta absoluta* (Meyrick) (Lepidoptera: Gelechiidae) originated in South America, and has been one of the worst pests in South America since the 1950s and can reduce crop yields by 80–100% [1,2,3]. It threatens crops in the nightshade family such as tomatoes, potatoes, eggplant, peppers, and tobacco [4,5]. *T. absoluta* mainly damages in the larval stage, and can do harm in any development stage and any above-ground part of the tomato plant. Since its accidental introduction to Spain in 2006, the pest has rapidly invaded many countries and regions, posing a serious threat to the global tomato production [2,6,7,8,9,10]. *T. absoluta* was reported in Israel in 2009, then spread steadily in 15 western Asian countries between 2010 and 2015, and has been found in several countries in southern, central, and eastern Asia in recent years. It can be seen that its invasion range is wide, and the invasion speed is fast [5]. The invasive species was firstly discovered in August 2017 in Huocheng County, Ili Kazakg Autonomous Prefecture, Xinjiang Uygur Autonomous Region (Huocheng, Ili, Xinjiang), China. The successful invasion of the pest has posed a great threat to the tomato industry in Xinjiang and other places [5]. So, it is particularly important and urgent to study its invasion mechanism. As an important parameter affecting population growth and development, temperature is closely related to successful invasion and colonization.

Temperature is an important factor limiting the adaptive distribution of species and determines the distribution and diffusion area of species. Insects are very sensitive to temperature changes [11,12]. In fact, temperature is one of the key abiotic factors that directly affects insect reproduction, development, activity, adaptation, survival, and spatio-temporal distribution [13,14]. In nature, insects are faced with multiple environmental pressures, which may seriously affect their survival. In order to adapt to the environment, insects can adopt a set of mechanisms on the acute and chronic timescales, so as to improve the adaptability in the suboptimal environment. Including *T. absoluta*, there was some research about the behavioral observations under different temperature environments [15,16,17], and the underlying molecular mechanism has not been declared. Li et al. [15] reported that that temperature affected the growth, development, and reproduction of *T. absoluta*. The developmental duration of *T. absoluta* was shortened with the increase in temperature in the range of 15–30 °C. The survival rate of the larval and the early adult, and the total egg production of the female moth reached the maximum at 25 °C. In addition, both the adults and the larvae of *T. absoluta* were chill susceptible and successfully overwintered in warm African environments. However, studies have shown that larvae are more resistant to cold than adults, which may be due to the advantages of the physiological adaptation and ecological factors of larvae [17]. Furthermore, Tarusikirwa et al. [16] revealed that the thermal plasticity of *T. absoluta* larvae is stronger than that of adults, and they can change their thermal tolerance in a short time and long timescales.

Body temperature (Tb) plays a key role in the performance of ectotherms [18,19,20,21], which regulate body temperature through behavioral responses in order to achieve the appropriate temperature conditions [22,23,24]. When presented with a choice of ambient temperatures, as in a thermal gradient, motile organisms tend to congregate in, or spend the most time in, a relatively narrow range of temperatures. Such behavior is termed temperature preference or behavioral thermoregulation [25,26,27,28]. At present, for more than 50 species of terrestrial invertebrates, researchers have measured the preferred temperature (Tpref), which is closely related to the ecological process from individual behavior to population and community dynamics as an indicator of behavioral temperature regulation in a variable environment [29,30,31,32]. In addition, the preferred temperature can be measured by a thermal gradient [19,26,27,28]. Insects, as small ectotherms, are very sensitive to temperature perception, and Tpref reflects the individual’s choice of the most favorable thermal microhabitat, which is related to increasing foraging opportunities and avoiding predators [33,34,35]. Quantifying the Tpref of individuals is, therefore, important in understanding how thermal landscapes mechanistically contribute to shaping the distributions of species via behavioral thermoregulation [29].

Transient receptor potential channels, also known as TRP channels, are a superfamily of ion channels occurring on cell membranes and are related to various sensory receptions, including thermal receptivity, chemical receptivity, mechanical receptivity, and light receptivity [36]. The channel was firstly identified in the late 1970s and early 1980s in the light-sensing cells of fruit flies [37]. Since then, these channels have been found in a variety of organisms from worms to flies and humans [38,39]. According to the similarity of gene sequences and protein structures, these channels have been divided into seven families, namely TRPC, TRPV, TRPA, TRPN, TRPM, TRPML, and TRPP [40,41]. These channels are located on the outer membranes of different types of sensory cells and respond to various stimuli by regulating the flow of potassium, calcium, and sodium plasma in and out of the cells [36]. Mammalian TRPV is a classical “thermoTRP”, which can be directly activated at temperatures greater than or equal to 42 °C [42]. *Drosophila* larvae and adults also use thermoTRPs to avoid harmful cold and heat, and to identify minute temperature differences within a comfortable range (18–24 °C) [36], and use the “thermoTRP” mechanism to identify the comfort zone. The noxious response is a necessary condition for animals to respond quickly to potentially fatal sensory attacks. There are at least three TRP channels in the adult *Drosophila melanogaster* that contribute to the timely response to overheating injury, namely TRPA1, Pyrexia (Pyx), and Painless (Pain), all of which belong to the TRPA subfamily [43,44]. When subjected to a harmful temperature (>39 °C) or strong mechanical stimulation, larvae made an evasive response dependent on mdIV neurons and TRPA1 and Painless (Pain) channels [44,45,46,47]. Pain has a temperature activation threshold of 39–42 °C and is expressed in mdIV neurons [45,48]. The Pyx channel is directly activated by high temperatures close to 40 °C, and, when exposed to 40 °C, a mutation in fever leads to faster paralysis [43]. The results show that TRP protein can be activated by specific temperature changes, so that neurons can convey temperature information and play the role of molecular thermometers in insects [49,50]. In the study of two cryptic species of *Bemisia tabaci* AsiaII3 and MEAM1 (Middle East Asia Minor 1), it was found that temperature stress could induce the expression of *BtTRP* mRNA, and played an important role in heat tolerance. According to this characteristic, researchers infer that *BtTRP* also belongs to the TRPA subfamily [51,52,53]. Therefore, the TRPA subfamily plays an important role in temperature sensing and the heat escape injury response.

To assess the preferred temperature sensation mechanism of the TRPA genes in *T. absoluta*, the following aspects were explored. First, we cloned partial or full-length cDNA sequences of the TRPA genes and analyzed the characteristics of these genes. Second, we examined the mRNA expression profiles of the *TaTRPA1*, *TaPain*, *TaPyx* genes during different developmental stages by quantitative real-time PCR. Third, we identified the function of the TRPA genes in the selection preference temperature using the feeding dsRNA method. Our findings can provide a theoretical basis for further study on the mechanisms of adaptation to environmental temperatures of *T. absoluta*.

## 2. Results

### 2.1. Cloning of TaPain and TaPyx

**Figure 1:**
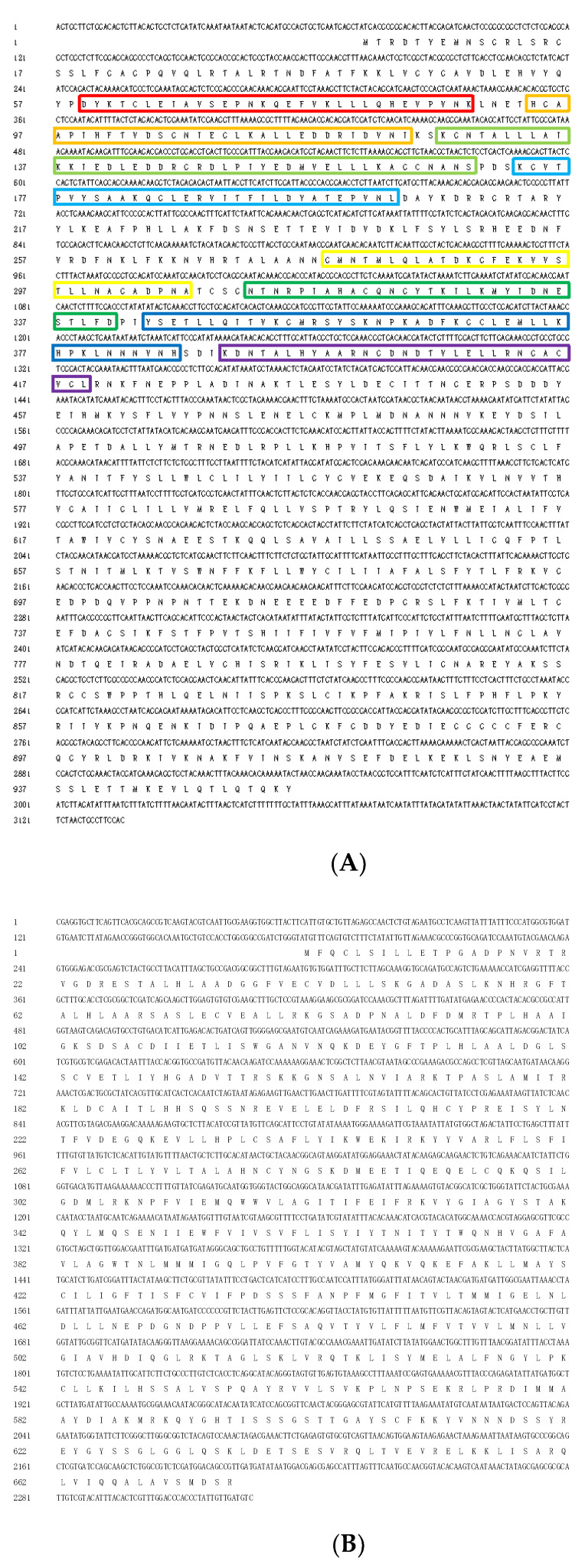
(**A**). Full-length cDNA sequence of *Tuta absoluta TaPain* and its deduced amino acid sequence. The full-length cDNA of *T. absoluta TaPain* is 3136 bp, and the open reading frame (72–2936 bp) encodes a polypeptide of 954 amino acids. Eight boxes in different colors are marked with eight ankyrin repeats. (**B**). Partial length cDNA sequence of *Tuta absoluta TaPyx* and its deduced amino acid sequence. The partial length cDNA of *T. absoluta TaPyx* is 2326 bp, and the open reading frame 178–2202 bp encodes a polypeptide of 674 amino acids.

The full-length cDNA of *T. absoluta TaPain* is 3136 bp and contains a 71-bp 5′-untranslated region (5′-UTR) (positions 1–71), a 200-bp 3′-UTR (positions 2937–3136), and a 2865-bp open reading frame (ORF) (positions 72–2936). The ORF encodes a polypeptide of 954 amino acids with a calculated molecular mass of 259.89 kDa and an isoelectric point (pI) of 4.86 (Figure 1A). The accession numbers of *TaPain* are MZ382839.

The partial length cDNA of *T. absoluta TaPyx* is 2326 bp and contains a 177-bp 5′-untranslated region (5′-UTR) (positions 1–177), a 124-bp 3′-UTR (positions 2203–2326), and a 2025-bp open reading frame (ORF) (positions 178–2202). The ORF encodes a polypeptide of 674 amino acids with a calculated molecular mass of 193.16 kDa and an isoelectric point (pI) of 4.92 (Figure 1B). The accession numbers of *TaPyx* genes are MZ382840.

### 2.2. Sequence Analysis of TaTRPA1, TaPain, and TaPyx

**Figure 2:**
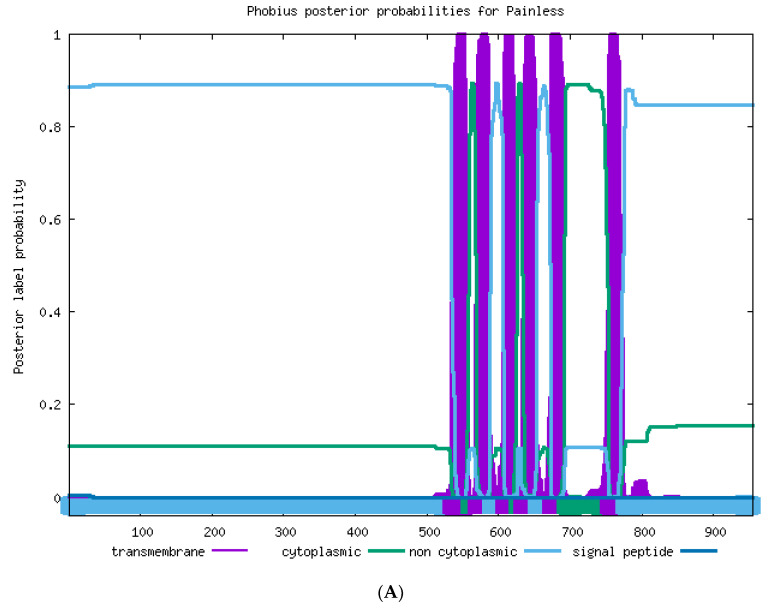

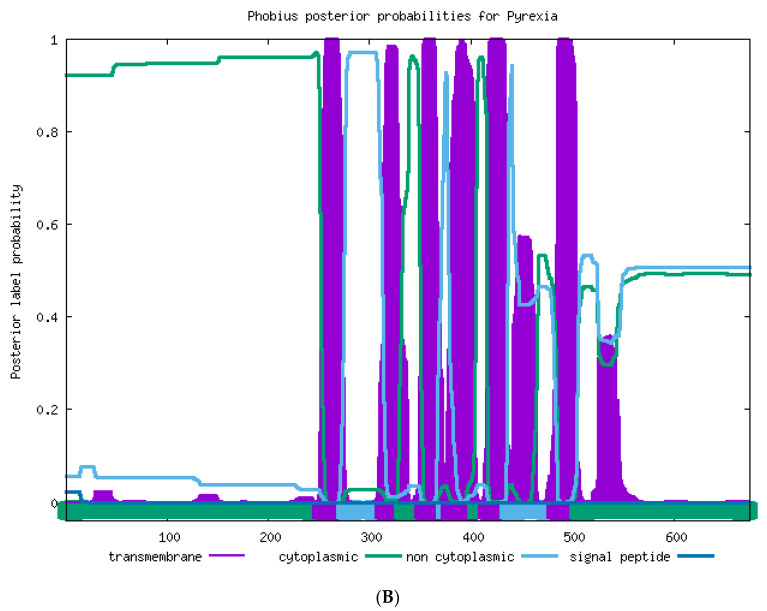
(**A**). Transmembrane structure prediction of the *TaPain* protein in *Tuta absoluta*. Six transmembrane structures were found in *Tuta absoluta TaPain*. The transmembrane structural positions of TM1, TM2, TM3, TM4, TM5, and TM6 were located at the amino acid positions of 535–558, 570–588, 608–626, 633–653, 673–692, and 754–775, respectively. (**B**). Transmembrane structure prediction of the *TaPyx* protein in *Tuta absoluta*. Six transmembrane structures were found in *Tuta absoluta TaPyx*. The transmembrane structural positions of TM1, TM2, TM3, TM4, TM5, and TM6 was located at the amino acid positions of 252–275, 314–332, 353–373, 379–404, 416–436, and 483–505, respectively.

The transmembrane helices in the TRPA protein were predicted by using the online software Phobius (https://phobius.sbc.su.se/. Accessed on 20 April 2021). As shown in Figure 2A, eight ankyrin repeats were found in *T. absoluta Painless*; the amino acid positions of ANK1–ANK8 were as follows: 59–89, 94–124, 127–169, 173–203, 278–307, 312–341, 344–386, 390–419, and six transmembrane structures (TM1–TM6) were found in *T. absoluta TaPain*, and the amino acid positions of 535–558, 570–588, 608–626, 633–653, 673–692, 754–775 indicated the transmembrane structure positions of TM1, TM2, TM3, TM4, TM5, and TM6, respectively. There are six transmembrane structures in *T. absoluta TaPyx* (Figure 2B). The amino acid positions of TM1–TM6 were as follows: 252–275, 314–332, 353–373, 379–404, 416–436, 483–505.

**Figure 3:**
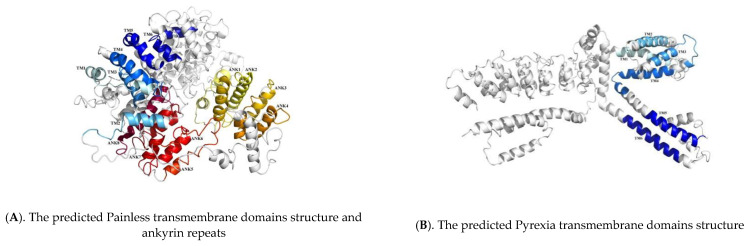
The conserved domains of Painless and Pyrexia in *Tuta absoluta*. The light blue to dark blue represent different transmembrane domains structure, and the light yellow to red represent different ankyrin repeats.

We also used Swiss-Model online software to predict the three-dimensional structures of Painless and Pyrexia, and found that they both contained six transmembrane domains, and found eight ankyrin repeats of Painless. It is helpful for us to understand the structural characteristics and functions of the protein through the three-dimensional structure of the protein. The specific spatial conformation formed by the folding of Painless and Pyrexia proteins allows us to see more visually the transmembrane domain and ankyrin repeats, as shown in Figure 3.

**Figure 4:**
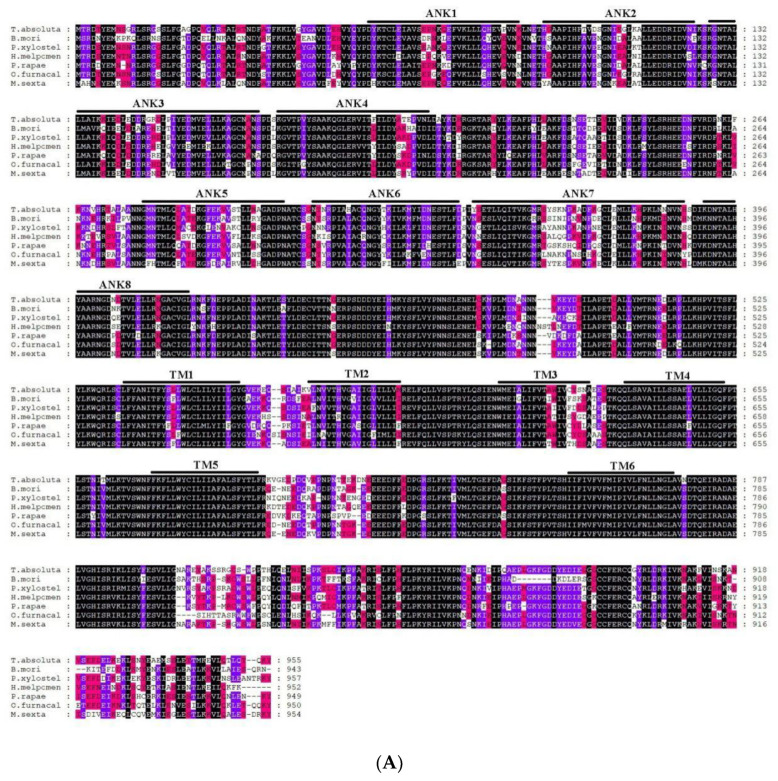

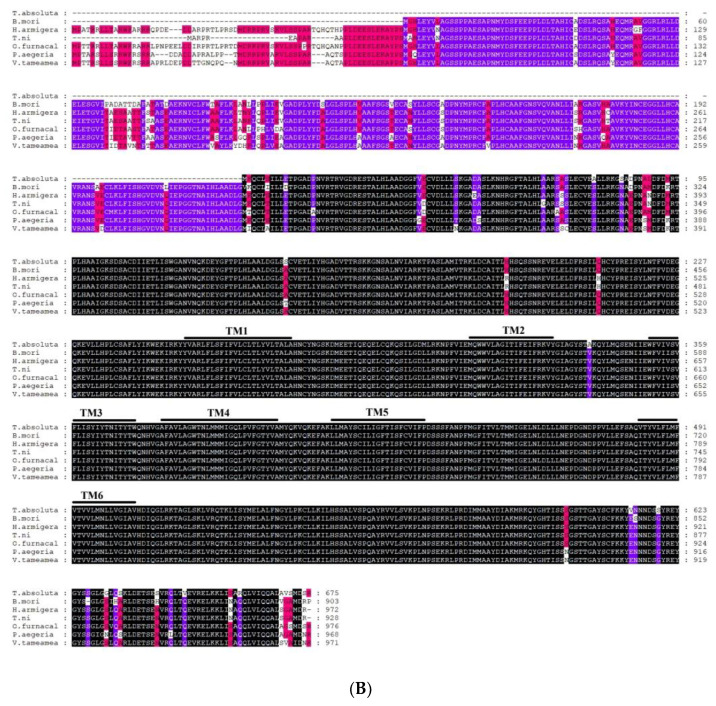

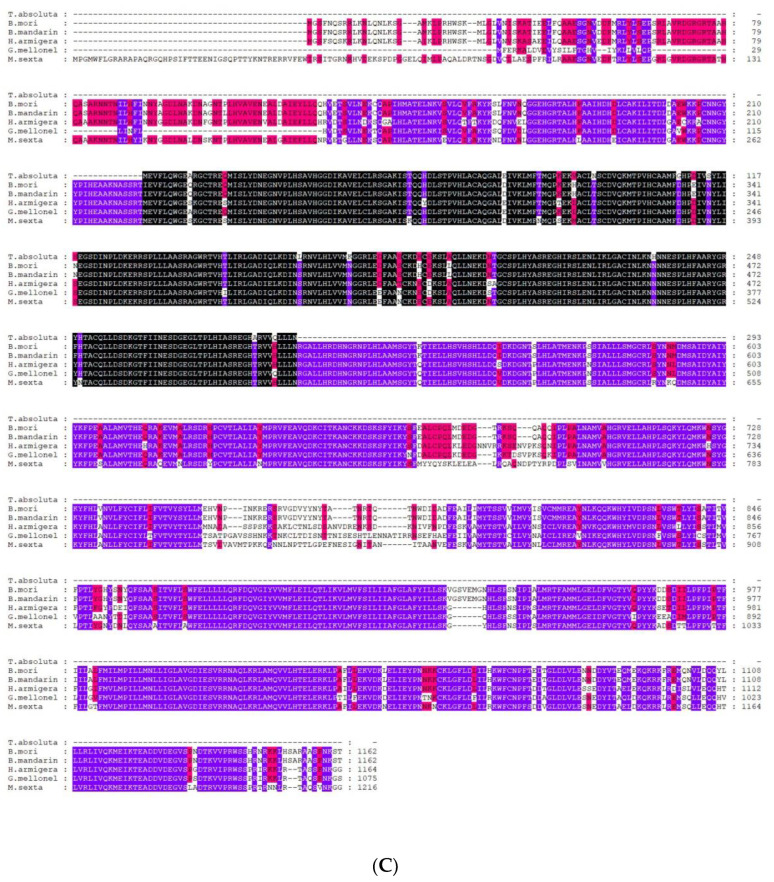
(**A**). Alignment of TRP protein from *Tuta absoluta* and other insects. Black represents that the amino acid sequences of seven species in multiple sequence alignment are completely identical; purple represents that six species out of seven species have the same amino acid sequences at this site; red represents that four to five species out of seven species have the same amino acid sequences at this site; and no color represents that there are differences among species. *B. mori*: *Bombyx mori* Painless (001296553.1); *P. xylostella*: *Plutella xylostella* Painless (XP_011560772.2); *H. Melpomene*: *Heliconius Melpomene* Painless (QDR50965.1); *P. rapae*: *Pieris rapae* Painless (XP_022120653.1); *O. furnacalis*: *Ostrinia furnacalis* Painless (XP_028177403.1); *M. sexta*: *Manduca sexta* Painless (XP_030023596.2). (**B**). Alignment of Pyrexia protein from *Tuta absoluta* and other insects. Black represents that the amino acid sequences of seven species in multiple sequence alignment are completely identical; purple represents that six species out of seven species have the same amino acid sequences at this site; red represents that four to five species out of seven species have the same amino acid sequences at this site; and no color represents that there are differences among species. *B. mori*: *Bombyx mori* Pyrexia (NP_001296553.1); *H. armigera*: *Helicoverpa armigera* Pyrexia (XP_021194189.1); *T. ni*: *Trichoplusia Pyrexia* ni(XP_026735810.1); *O. furnacalis*: *Ostrinia furnacalis* Pyrexia (XP_028163008.1); *P. aegeria*: *Pararge aegeria* Pyrexia (XP_039759808.1); *V. tameamea*: *Vanessa tameamea* Pyrexia (XP_026483194.1). (**C**). Alignment of TRPA1 protein from *Tuta absoluta* and other insects. Black represents that the amino acid sequences of six species in multiple sequence alignment are completely identical; purple represents that five species out of six species have the same amino acid sequences at this site; red represents that four species out of six species have the same amino acid sequences at this site; and no color represents that there are differences among species. *B. mori*: *Bombyx mori* TRPA1 (NP_001296525.1); *B. mandarina*: *Bombyx mandarina* TRPA1(XP_028033887.1); *H. armigera*: *Helicoverpa armigera* TRPA1(XP_021185779.1); *G. mellonella: Galleria mellonella* TRPA1(XP_031767554.1); *M. sexta: Manduca sexta* TRPA1(XP_037299698.1).

A blastx program showed that *T. absoluta Painless* shared more than 73% identity with previously identified Painless proteins from other *Lepidoptera* (*Bombyx mandarina* XP_028039162.1; *Bombyx mori* NP_001296553.1; *Danaus plexippus plexippus* XP_032511715.1; *Helicoverpa armigera* XP_021191851.1; *Zerene cesonia* XP_038213738.1; *Trichoplusia ni* XP_026747274.1; *Spodoptera litura* XP_022826996.1; *Pararge aegeria* XP_039745767.1; *Operophtera brumata* KOB66914.1; *Papilio Xuthus* KPJ01914.1; *Vanessa tameamea* XP_026491621.1; *Ostrinia furnacalis* XP_028177403.1; *Heliconius Melpomene* QDR50965.1; *Pieris rapae* XP_022120653.1; *Manduca sexta* XP_030023596.2; *Plutella xylostella* XP_011560772.2.). We directly compared the amplified *TaTRPA1* nucleotide sequence to blastx sequences in NCBI, and it was found that the identity with known *TRPA1* sequences among 26 species, such as *M*. *Sexta* and *Spodoptera* *frugiperda*, exceeded 75%. The identity of *TaPyx* with known *Pyx* sequences among 43 species, such as *B*. *mori* and *H*. *armigera*, was over 70%. We selected six representative species for multiple sequence alignment, and found that the identity of the Painless protein was up to 84.57%, and that of the Pyrexia protein was up to 89.78%. Although TRPA1 only amplified part of the sequences, the identity between the amplified sequences and the other five species was 70.96%. The results of multiple sequence alignment are shown in Figure 4A–C, which indicated that these three genes were all relatively conserved.

**Figure 5:**
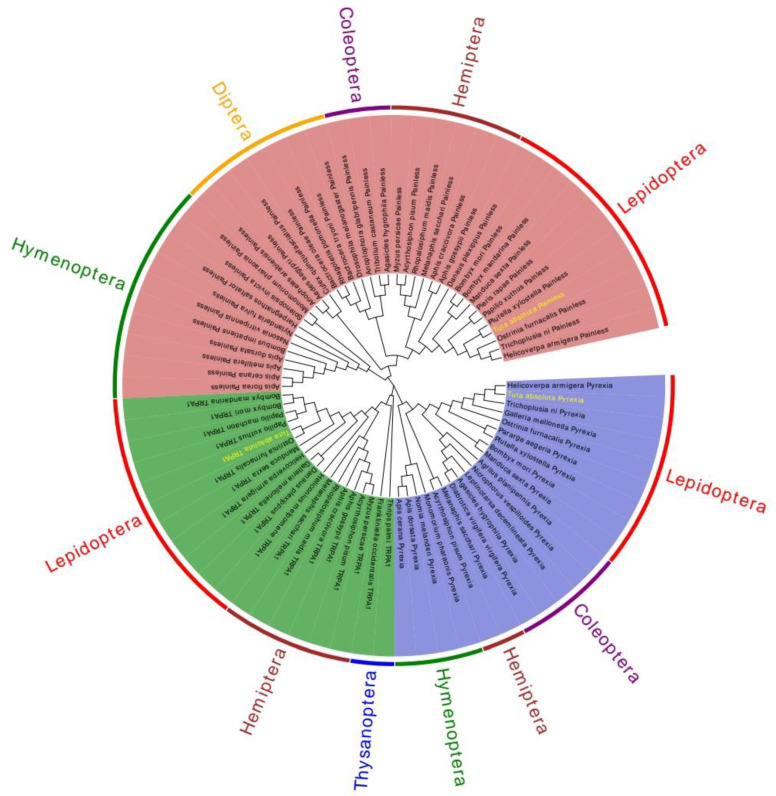
A phylogenetic tree based on the known amino acid sequences of TRPA genes. The phylogenetic tree was generated via Maximum Likelihood method based on the poisson correction mode, and this tree was used to determine the relationships between different insects. *Helicoverpa armigera* Pyrexia (XP_021194189.1); *Trichoplusia ni* Pyrexia (XP_026735810.1); *Galleria mellonella* Pyrexia (XP_026761448.2); *Ostrinia furnacalis* Pyrexia (XP_028163008.1); *Pararge aegeria* Pyrexia(XP_039759808.1); *Plutella xylostella* Pyrexia(XP_037962898.1); *Bombyx mori* Pyrexia (NP_001296536.1); *Manduca sexta* Pyrexia (XP_030021352.2); *Agrilus planipennis* Pyrexia (XP_018334781.1); *Nicrophorus vespilloides* Pyrexia (XP_017775114.1); *Leptinotarsa decemlineata* Pyrexia (XP_023020123.1); *Agasicles hygrophila* Pyrexia (QLH02045.1); *Diabrotica virgifera virgifera* Pyrexia (XP_028131629.1); *Melanaphis sacchari* Pyrexia (XP_025205608.1); *Acyrthosiphon pisum* Pyrexia (XP_016657902.1); *Monomorium pharaonic* Pyrexia (XP_012535072.2); *Nomia melanderi* Pyrexia (XP_031825641.1); *Apis dorsata* Pyrexia (XP_006623313.1); *Apis cerana* Pyrexia (XP_028522925.1); *Thrips palmi* TRPA1 (XP_034255303.1); *Frankliniella occidentalis* TRPA1 (XP_026285236.1); *Myzus persicae* TRPA1 (XP_022167295.1); *Acyrthosiphon pisum* TRPA1(XP_029342925.1); *Aphis gossypii* TRPA1 (XP_027848279.1); *Aphis craccivora* TRPA1 (KAF0770012.1); *Rhopalosiphum maidis* TRPA1 (XP_026809343.1); *Melanaphis sacchari* TRPA1 (XP_025200861.1); *Heliconius melpomene* TRPA1(QDR50963.1); *Danaus plexippus* TRPA1 (QDQ16924.1); *Galleria mellonella* TRPA1 (XP_031767554.1); *Helicoverpa armigera* TRPA1 (XP_021185779.1); *Manduca sexta* TRPA1 (QDR51038.1); *Ostrinia furnacalis* TRPA1 (XP_028170510.1); *Papilio xuthus* TRPA1 (KPJ00566.1); *Papilio machaon* TRPA1 (KPJ09099.1); *Bombyx mori* TRPA1 (NP_001296525.1); *Bombyx mandarina* TRPA1(XP_028033887.1); *Apis florea* Painless (XP_031772455.1); *Apis cerana* Painless (XP_028520736.1); *Apis mellifera* Painless(XP_006562517.1); *Apis dorsata* Painless (XP_031369976.1); *Bombus impatiens* Painless (XP_024223159.1); *Nasonia vitripennis* Painless (XP_031783731.1); *Nylanderia fulva* Painless (XP_029174885.1); *Harpegnathos saltator* Painless (XP_025162136.1); *Solenopsis invicta* Painless (XP_039303762.1); *Monomorium pharaonis* Painless (XP_036145416.1); *Anopheles arabiensis* Painless (XP_040153163.1); *Aedes aegypti* Painless (XP_001652261.2); *Culex quinquefasciatus* Painless (XP_001849122.2); *Bactrocera oleae* Painless (XP_036223549.1); *Rhagoletis pomonella* Painless(XP_036331109.1); *Bactrocera tryoni* Painless (XP_039969281.1); *Drosophila melanogaster* Painless(NP_611979.1); *Anoplophora glabripennis* Painless(XP_018573376.1); *Tribolium castaneum* Painless (NP_001164308.1); *Agasicles hygrophila* Painless (QLH02046.1); *Myzus persicae* Painless (XP_022174286.1); *Acyrthosiphon pisum* Painless (XP_029346868.1); *Rhopalosiphum maidis* Painless (XP_026806747.1); *Melanaphis sacchari* Painless (XP_025190493.1); *Aphis craccivora* Painless (KAF0773934.1); *Aphis gossypii* Painless (XP_027846115.1); *Danaus plexippus* Painless (QDQ16926.1); *Bombyx mori* Painless (NP_001296553.1); *Bombyx mandarina* Painless (XP_028039162.1); *Manduca sexta* Painless (XP_030023596.2); *Pieris rapae* Painless (XP_022120653.1); *Papilio xuthus* Painless (KPJ01914.1); *Plutella xylostella* Painless (XP_011560772.2); *Ostrinia furnacalis* Painless (XP_028177403.1); *Trichoplusia ni* Painless(XP_026747274.1); *Helicoverpa armigera* Painless (XP_021191852.1).

The phylogenetic tree was constructed using the maximum likelihood method with 1000 bootstrap replications in MEGA 7.0 software. By constructing phylogenetic tree analysis, the evolutionary history of these genes can be easily understood. Insects of the same order gather in the same branch, indicating that these genes are relatively conserved during the whole evolutionary process. The phylogenetic tree revealed that the TRPA1 (green region), Painless (blue region), and Pyrexia (red region) proteins of insects clustered on a single branch in each order, and eventually all the three proteins clustered on the same branch (Figure 5). In addition, it could be seen that the TRPA1 of *T. absoluta* and the *Lepidoptera* such as *O. furnacalis*, *P. xuthus*, *Papilio machaon*, *B. mori*, and *B. mandarina* clustered in the same branch. The Painless of *T. absoluta* and the *Lepidoptera* such as *P. xylostella*, *O. furnacalis*, *T. ni*, and *H. armigera* clustered in the same branch. The Pyrexia of *T. absoluta* and the *Lepidoptera* such as *H. armigera*, *T. ni*, *Galleria mellonella*, and *O. furnacalis* clustered in the same branch. The above results showed that that these genes are conserved throughout evolution, which is consistent with the traditional taxonomy.

### 2.3. Expression Profiles of TaTRPA1, TaPain, and TaPyx during Different Developmental Stages

**Figure 6:**
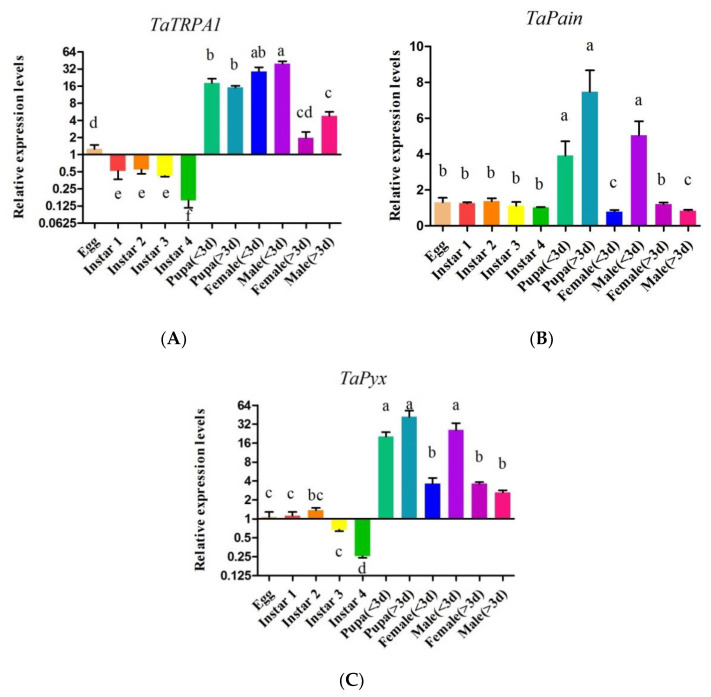
Relative expression levels of *TaTRPA1* (**A**), *TaPain* (**B**), *TaPyx* (**C**) in eggs, first to fourth instars, early to late pupae, newly emerged to mature females and males. Data represent means ± SEM. Bars with different lowercase letters are significantly different at *p* < 0.05.

Real-time PCR was used to isolate and amplify cDNA from eggs, nymphs from the first to fourth instars, early to late pupae, newly emerged to mature females and males. The results indicated that *TaTRPA1*, *TaPain*, and *TaPyx* expressed at all the developmental stages tested (Figure 6). The expression level of *TaTRPA1* in the pupal stage (39.81 ± 4.04, *df* = 4, *p* < 0.05) and newly emergence stage (29.17 ± 5.23, *df* = 4, *p* < 0.05) was significantly higher than that in other stages. The expression levels of Painless and *Pyrexia* in the pupal stage (7.48 ± 1.20, *df* = 4, *p* < 0.05; 41.96 ± 10.60, *df* = 4, *p* < 0.05) and incipient emerging males (5.04 ± 0.80, *df* = 4, *p* < 0.05; 25.80 ± 7.47, *df* = 4, *p* < 0.05) were significantly higher than those in other developmental stages. Through gene expression profile analysis, it was also found that: there was no significant difference in *TaTRPA1* between females and males, but there was a significant difference between newly emerged adults and mature adults. The relative expression level of *TaPain* in newly emerged males was significantly higher than that in females (5.04 ± 0.80, *p* < 0.05). Similarly, the relative expression level of *TaPyx* in newly emerged males was significantly higher than that in females (25.80 ± 7.47, *p* < 0.05). In addition, there was no significant difference in the expression levels of the three genes during the first, second, and third instar larvae.

### 2.4. The Function of TaTRPA1, TaPain, and TaPyx Genes in Temperature Preference Behavior

**Figure 7:**
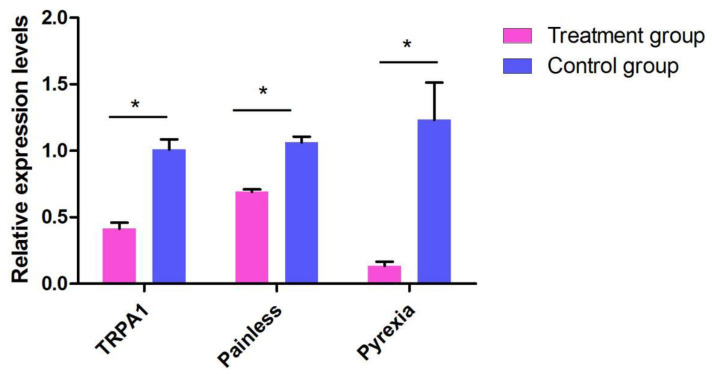
Effects of dsRNA treatments on mRNA expression in the *Tuta absoluta*. Data are presented as means ± SEM. Data were compared by analysis of variance (ANOVA) followed by Tukey’s post hoc test (* *p* < 0.05).

To investigate the role of TRPA in preference temperature further, *T. absoluta* were fed dsRNA to silence *TaTRPA1*, *TaPain*, and *TaPyx* gene expression, respectively. The mRNA expression after RNAi were significantly decreased compared to the control group, as shown in Figure 7. The *TaTRPA*1 mRNA expression level was 58.52% lower than that in the control group (ds*EGFP*), and the gene expression levels of *TaPain* and *TaPyx* were decreased by 30.95% and 86.62%, respectively.

**Figure 8:**
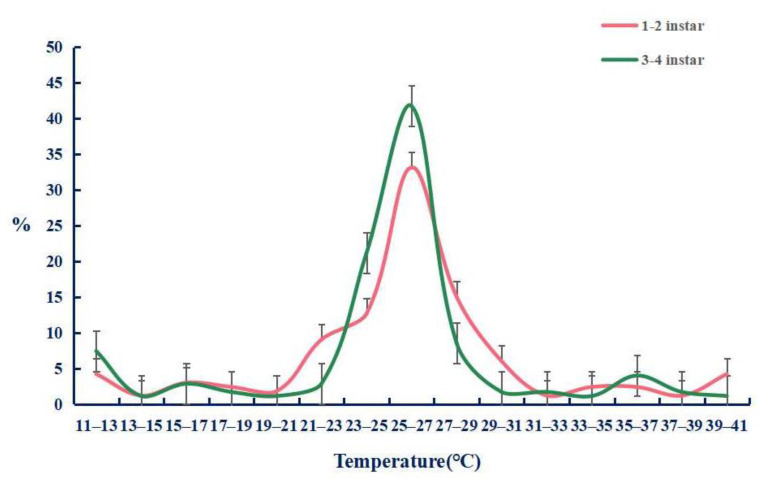
Temperature preference responses of *Tuta absoluta* larvae. The data are presented as mean ± SEM.

**Figure 9:**
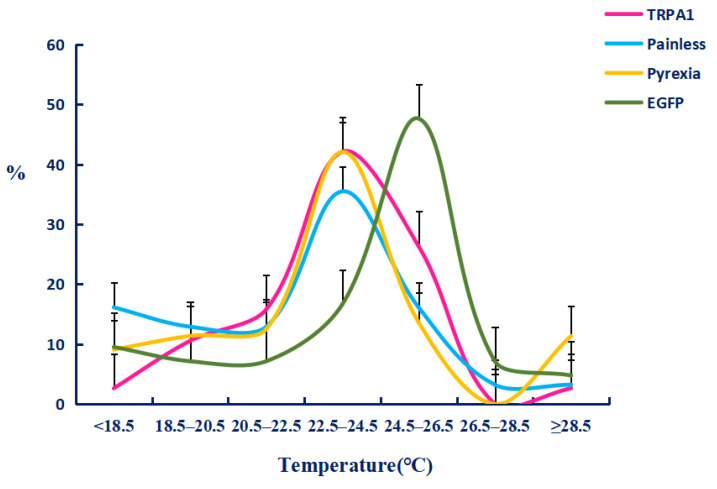
Temperature preference responses after dsRNA feeding in *Tuta absoluta* larvae. The data are presented as mean ± SEM.

We firstly tested the preference temperature of the 1 to 2 and 3 to 4 instar larvae raised in the greenhouse without any treatment, and found that their preferred temperature was 25–27 °C (Figure 8) with the percentages of 33.1 ± 3.4% and 41.9 ± 3.9%. On this basis, we tested the preference temperature after feeding ds*TRPA1*, ds*Pain*, ds*Pyx*, and control ds*GFP* (Figure 9). The experimental results showed that the preference temperature of feeding ds*TRPA1*, ds*Pain*, and ds*Pyx* was between 22.5 °C and 24.5 °C, which was about 2 °C lower than that of the control group, with the percentages of 44.4 ± 5.6%, 34.8 ± 6.2%, and 41.9 ± 1.2%, respectively, and was significantly lower than the control group. These results indicated that the preference temperature of the moth was changed after the interference of TRPA genes, and the preference temperature showed a decreasing trend.

## 3. Discussion

In this study, we obtained the full-length sequence of the *T. absoluta Painless* gene, and bioinformatics analysis revealed that it had eight ankyrin repeats and six transmembrane domains. Ankyrin repeats are about 33 amino acids in length and have at least four contiguous copies that are involved in protein-protein interactions. Interestingly, there are eight ankyrin repeats in *T. absoluta* but less in other reported insects [52,54]. According to the reported genome sequence of *Tribolium castaneum* [54], the Painless protein of *T. castaneum* was found to have six ankyrin repeats by SMART analysis, and *TcPain* played a role in the rapid acclimation to high temperature [55]; while only two ankyrin repeats were found in the TRP study of *B. tabaci*, and *BtTRP* played a role in thermal tolerance at 35 °C [52]. It can be calculated that the number of anchors varies greatly among different species. The ankyrin repeats have been found in proteins of diverse functions such as ion transporters and signal transducers [56,57]. However, the role of different amounts of ankyrin repeats in temperature adaptation needs to be further studied. In addition, it was found that Pyrexia had six transmembrane domains in *T. absoluta*, which is consistent with the results of previous studies on the TRPA subfamily. Besides, as shown in Figure 3B, there were two regions of low complexity detected by the SEG program. The function of these regions needs to be further studied, and it was speculated that they had little influence on the function of the whole Pyrexia. In previous studies, it was believed that the TRP channel usually contains four subunits, each of which has six transmembrane proteins, and its N-terminal and C-terminal are both intracellular [58], which is consistent with the protein prediction results of the *TaPyx* protein. However, studies in invertebrates have also found that the N-terminal and C-terminal of the TRP channel exist extracellular, such as the TRP channel in *B. tabaci* [51]. In fact, TRP is a class of nonselective cationic channel proteins that exist on the membrane of the cell membrane or intracellular organelle membrane, and the mechanism of its signal conversion in many systems needs to be further studied.

The results of the study based on the expression profile showed that there were differences in expression between females and males. Differential gene expression between the sexes leads to phenotypic differences between the sexes [59,60], which are manifested as differences in morphology, behavior, and physiology [61]. In the present study, there were also expression differences between females and males in *T. absoluta*, and their effects on behavior and physiology need to be further studied. In addition, the relative expression levels of *TaPain* and *TaPyx* at the initial emergence stage were significantly higher than those at the older development stage in males, which was also found in other insects such as *Monochamus alternatus* and *Frankliniella occidentalis* [62,63]. This phenomenon is common in invertebrates and may be a response to the transition from the larval stage to adult stage of insects through self-regulation to adapt to the environment.

There are many different sensory organs that can distributed in different tissues in *Drosophila* [36], and they can variously express such as in this present study. The expression levels of *TaTRPA1* and *TaPyx* were significantly lower in larvae than those in adults. Many studies showed that the cold resistance and thermal plasticity of larvae were higher than that of adults [16,17]. Therefore, we speculated that the larvae would be more sensitive to the selection of preference temperature, and the larvae were easy to perform the RNAi experiment, so we used larvae as the research objects.

Temperature preference is an important factor in understanding individual thermoregulation, population, or community dynamics. Here, we used the temperature preference meter to determine the preference temperature of *T. absoluta* quantitatively. In the process of recording data, dead or inactive insects were removed and not counted in the total, thus reducing experimental errors. The results of the study found that the preference temperature of *T. absoluta* is close to the suitable temperature for growth and development (25–27 °C), which is similar to wild-type *D. melanogaster* (~24 °C) [64]. In addition, it is consistent with the research results of *Apolygus lucorum* (25–28 °C) [65]. These studies show that insects have the ability to sense and respond to temperature changes. On this basis, by interfering with *TaTRPA*1, *TaPain*, and *TaPyx* genes, the temperature preference behavior of *T. absoluta* changed, and their preference temperature showed a decreasing trend. After interfering with the three target genes, the preferred temperature became 22.5–24.5 °C, which was 2 °C lower than the control group. The expression levels of *TaPain*, *TaPyx*, and *TaTRPA1* genes were decreased to different degrees by RNAi, and the preferred temperature was changed to different degrees after interference, and the final result was two degrees lower than the optimal temperature of the control group, suggesting that these genes play a similar role in the selection of temperature preference of *T. absoluta*. *DmTRPA1* was helpful for *D. melanogaster* larvae to choose an 18 °C environment as much as possible [66], which was lower than the suitable temperature for growth and development. Similarity, Dillon et al. reported that knocking out related genes resulted in a decrease in preference temperature in ectotherms [67]. As a temperature sensitive element, *DmTRPA1* can not only sense the change rate of external temperature [68], but also regulate the physiological rhythm of *D. melanogaster* [69]. *DmPyx* can also regulate the biological clock of *D. melanogaster* by sensing temperature [70]. *BmTRPA1* can not only make silkworms sense the temperature change and make an immediate response quickly, but also regulate the long-term adaptive diapause response related to the temperature and affect the diapause behavior of offspring [71]. The interference results showed that *AgTRPA1* could regulate the preference of larvae to a higher temperature [72]. *TcTRPA1*, *TcPain*, and *TcPyx* also played an important role in the heat tolerance of *T. castaneum*. The silence of *TcTRPA1* affected its escape behavior to high temperature (39 and 42 °C) [55].

## 4. Materials and Methods

### 4.1. Insect Rearing and Host Plants

The tomato leaf miner *Tuta absoluta* colony used in this experiment was originally collected in Yuxi, Yunnan Province, in August 2018. The tomato variety planted is Maofen. In the laboratory, the tomato leaf miner was reared in an insectary at 25 ± 2 °C under 50–60% relative humidity with a 14:10 h light:dark cycle. The host plants were individually grown in 9-cm-diameter pots under the same conditions as the tomato leaf miner.

### 4.2. RNA Extraction and cDNA Synthesis

Total RNA was isolated using the Micro total RNA Extraction Kit (Tianmo Biotech, Beijing, China). Subsequently, a NanoPhotometerTM P330 (Implen, Munich, Germany) and 1% agarose gel electrophoresis were used to determine the RNA quality and concentration. The first-strand cDNA was generated from 1.0 μg RNA using the Super Script First-Strand Synthesis System (TransGen, Beijing, China).

### 4.3. Cloning of the Three Genes of TRPA Family

The full length cDNAs were obtained using a Taq DNA Polymerase amplification kit (TransGen, Beijing, China) according to the manufacturer’s instructions. The *TRPA1*, *Painless*, and *Pyrexia* homologous genes of *B. mori* (BAO53207.1, BAO53208.1, and NP_001296536.1) and *D. melanogaster* (NP_001261602.1, NP_001261176.1, and NP_612015.1) were used to query the transcriptome dataset of *T. absoluta* by blastp and tblastn. According to the corresponding sequences of *T. absoluta*, primers were designed by primer 5.0 (Table 1). The amplified fragments were purified using an AxyPrep TM DNA Gel Extraction Kit (Axygen, West Orange, NJ, USA). Finally, the distinct single-band amplification products were cloned into the pEASY-T3 vector (Transgen) and sequenced.

### 4.4. Sequence Analysis of the Three TRPA Genes

Sequence alignment and identity analyses were performed using DNAMAN (version 5.0; LynnonBioSoft, QC, Canada). Molecular weights and pIs were calculated using ExPASy (http://web.expasy.org/protparam/. Accessed on 20 April 2021). Conserved functional domains of the deduced protein sequences of the three genes were identified using SMART software (http://smart.embl-heidelberg.de/. Accessed on 20 April 2021). Crossmembrane domains was predicted using TMHMM Server v. 2.0 (http://www.cbs.dtu.dk/services/TMHMM/. Accessed on 20 April 2021). Multiple protein sequences were aligned using DNAMAN and implemented in the MAGE 7.0 software package to evaluate the molecular evolutionary relationships between TRPA1, Painless and Pyrexia genes, and various insects. The amino acid sequences of TRPA1, Painless, and Pyrexia of different insects were downloaded from NCBI (https://www.ncbi.nlm.nih.gov/. Accessed on 1 May 2021) to construct the phylogenetic tree. The phylogenetic tree was constructed with the maximum likelihood method using MAGE 7.0 software. Bootstrap majority consensus values for 1000 replicates are indicated at each branch point (%).

### 4.5. Quantitative Real-Time PCR Analysis of Relative Expression Levels

The expression profiles in different development stages and the effect of gene silencing after feeding dsRNA in *T. absoluta* were assessed. The different development stages included eggs, nymphs from the first to fourth instars, early to late pupae, newly emerged to mature females and males. The relative mRNA expression level was analyzed by reverse transcription real-time polymerase chain reaction. The primer sequences used are listed in Table 1. The reactions were performed using an ABI 7500 Real-time PCR system (Applied Biosystems, Waltham, MA, USA). All amplifications were confirmed by sequencing, and the specificity of RT-qPCR reactions was estimated by melting curve analysis. PCR assays were prepared to a final volume of 20.0 μL with 1.0 μL of the cDNA template, 10.0 μL of 2× TransStart TM Green qPCR SuperMix (Transgen), 200 μM of each gene-specific primer (Table 1), and 0.4 μL of Passive Reference Dye (Transgen). A thermocycler was programmed with the following cycling conditions: (1) 94 °C for 1 min, followed by (2) 40 cycles of 95 °C for 15 s, 61 °C for 30 s, and 72 °C for 30 s. There were three repetitions for each treatment or control, with 4 larvae in each repetition, and each repetition was assessed in triplicate (technical replicates). RpL5 (large subunit 5 ribosomal protein) was used as the reference gene. Amplification efficiency was validated by constructing a standard curve using seven serial dilutions of cDNA. The relative quantification of mRNA expression was calculated using the mathematical model of (Livak and Schmittgen 2001; Pfaffl 2001) [73,74], which simplifies to 2^−ΔΔCT^ as follows: (ΔΔCT = (Ct target − Ct reference) treatment − (Ct target − Ct reference) control).

### 4.6. Production of dsRNA Transcription Templates and Synthesis of dsRNA

To generate dsRNA, three fragment templates of *TRPA1*, *Painless*, and *Pyrexia* were amplified by PCR using cDNAs cloned previously as templates with forward and reverse primers containing the T7 primer sequence (Table 1) at the 5′ ends, respectively. Amplification reactions were conducted in 25 μL containing 19.0 μL of ddH_2_O, 2.5 μL of 10 × buffer, 0.5 μL of dNTPs (10 mM for each nucleotide), 1.0 mL of forward primer (10 mM/μL), 1.0 mL of reverse primer (10 mM/μL), 0.5 μL of cDNA template, and 0.5 μL of Taq DNA Polymerase (5 UμL^−1^; TransStart). The PCR cycling conditions were as follows: 94 °C for 5 min, followed by 35 cycles of 94 °C for 30 s, 60 °C for 30 s, and 72 °C for 30 s, and a final extension step of 72 °C for 10 min. The amplification of PCR products was confirmed by separation on 1.5% agarose gels and visualized by staining with ethidium bromide under UV light. The sequences were verified by sequencing of Sangon Biotech. dsRNA was synthesized using the MEGAscript RNAi Kit (Ambion, Austin, TX, USA), and 1 μg of PCR product was used as the transcription template. dsRNA was resuspended in RNasefree water. dsRNA was analyzed by agarose gel electrophoresis and quantified spectrophotometrically. The dsRNA was stored at −80 °C prior to further use.

### 4.7. RNA Interference (RNAi) and Detection

In this experiment, double-stranded RNA was delivered to *T. absoluta* larvae via feeding. Detached leaflets from Maofen tomatoes had their petioles immersed in 200 μL of water containing either 5 μg of dsRNA from each target gene or a GFP control, in triplicate. Uptake of the dsRNA solution by the tomato leaflets took 3–4 h. Immediately after uptake, second instar larvae (n = 15) were gently placed onto leaflets for feeding, and individuals were sampled 48 h after initiation of feeding. Controls with dsRNA from the GFP gene sequence were run in parallel. The effects of RNAi on the larvae were evaluated by comparative quantification real-time PCR of each target gene compared to the control.

### 4.8. Preference Temperature Behavioral Assay

A temperature preference tester was used to test the preference temperature of the tomato leaf miner. By using the semiconductor refrigeration chip as the temperature control unit, the temperature can be quickly and accurately controlled. The tester has a flat plate (30 cm × 10 cm × 1 cm) capable of generating a range of temperature gradients. The flat plate in the test area is 0.72 °C/cm. There is a 0.4 cm high plexiglass cover above the plate to prevent the larvae from escaping and to ensure that they can move freely on the temperature plate [28]. In each experiment, 15 s instar larvae were placed in the temperature range of 16–30 °C and allowed to move for 20 min under dark conditions [75], taking pictures every 10 min to observe and record the distribution of *T. absoluta* on the temperature plate. By observing and counting the number of larvae staying in different temperature zones, the percentage of the number of larvae in the temperature zone was finally calculated. These percentages represent the degree to which *T. absoluta* prefers the temperature. Each treatment had 4 biological replicates.

### 4.9. Statistical Analysis

Statistical analyses were carried out using the GraphPad Prism 5.0 (GraphPad, San Diego, CA, USA). The target gene expression profile was analyzed using one-way analysis of variance (ANOVA), followed by Tukey’s multiple comparison test. The RNA interference efficiency and the repressed gene expression level was analyzed by t test. Data are presented as mean ± standard error (mean ± SEM). Differences were considered significant when *p* < 0.05. The temperature preference behavior after RNAi was calculated by the percentage of larvae in the total number in each temperature zone.

## 5. Conclusions

In summary, the present study firstly indicated the molecular characterization of *TaPain* and *TaPyx* in *T. absoluta*, and the mRNA expression profile of *TaTRPA1*, *TaPain*, and *TaPyx* during different developmental stages. The results of feeding *TaTRPA1*, *TaPain*, and *TaPyx* dsRNA showed that the three target genes were key elements in temperature perception and played a key role in preference temperature. Our data improve our understanding of the mechanism of temperature sensation at the molecular level in *T. absoluta*, and provide some basis for the study of other *Lepidoptera* insects’ temperature preference. Moreover, it is of great significance in enriching the research progress of “thermos TRP”.

## Figures and Tables

**Table 1:** Primers used for cDNA cloning, quantitative real-time PCR (qPCR), double-stranded RNA (dsRNA) synthesis.

Gene Name	Primer Name	Primer Sequence (5´→3´)	Amplicon Length/bp
Primers for full-length gene amplification			
TRPA1	TRPA1-F277	GCGGTGGAGTTGTGCTT	515
	TRPA1-R791	CGGCTGGCGTAATGTAG	
Painless	Pain-F139	AGTGCTTGTGGACAGTGTT	1488
	Pain-R1626	CTAGCGAGTTATTTGGGTAA	
	Pain-F1456	GCGTCGGACTACGAAATA	1314
	Pain-R2769	GCAGAAAGTGAGGAAACAA	
	Pain-F2546	ACAAGAGATAAGAGCGGATG	727
	Pain-F3272	GTGGAAGGCAGTTAGAAGTA	
Pyrexia	Pyx-F1	CGAGGTGCTTCAGTTCA	1250
	Pyx-R1250	CTCACGATTACAAACCATTC	
	Pyx-F867	AGTGCTTTTACATCCG	1460
	Pyx- R2326	GACATCAACAATAGGGT	
Primers for qPCR			
TRPA1	TRPA1-QF123	ACACGAAGCAGCCAAAAACG	183 (for RNAi)
	TRPA1-QR305	GCTCCGGACCTCAAGCACAA	
	TRPA1-QF675	AGGAGGGAGGTTGGAAGAC	115 (for expression)
	TRPA1-QR789	GCTGGCGTAATGTAGAGGC	
Painless	Pain-QF2749	GTTTGTTTCCTCACTTTCTGCC	132
	Pain-QR2880	ATCCACCGCCTTCTATATCCTC	
Pyrexia	Pyx-QF1821	TTCTTCTGCCCTTGTCTCACC	135
	Pyx-QR1955	CCGTATTGTTTCCGCATTTTG	
RpL5	RpL5-QF	CAGTCGTCGAGCCAGCAACA	129 bp
	RpL5-QR	TCCCGCATTGAAGGAGACCA	
Primers for dsRNA synthesis			
TRPA1	TRPA1-DF277	taatacgactcactatagggGCGGTGGAGTTGTGCTT	515
	TRPA1-DR791	taatacgactcactatagggCGGCTGGCGTAATGTAG	
Painless	Pain-DF1456	taatacgactcactatagggGCGTCGGACTACGAAATA	303
	Pain-DR1758	taatacgactcactatagggTCAGAAGTGGTCGCAAAT	
Pyrexia	Pyx-DF1281	taatacgactcactatagggCATCACGTACACATGGCAAAA	335
	Pyx-DR1615	taatacgactcactatagggAGAACTCAAGTAAAACGGGGG	
